# Inverse association between dengue, chikungunya, and Zika virus infection and indicators of household air pollution in Santa Rosa, Guatemala: A case-control study, 2011-2018

**DOI:** 10.1371/journal.pone.0234399

**Published:** 2020-06-19

**Authors:** Zachary J. Madewell, María Reneé López, Andrés Espinosa-Bode, Kimberly C. Brouwer, César G. Sánchez, John P. McCracken

**Affiliations:** 1 Centro de Estudios en Salud, Universidad del Valle de Guatemala, Guatemala City, Guatemala; 2 PhD Program in Public Health (Epidemiology), University of California, San Diego, CA, United States of America; 3 San Diego State University, San Diego, CA, United States of America; 4 Division of Global Health Protection, Centers for Disease Control and Prevention, Atlanta, GA, United States of America; 5 Division of Global Health, Department of Family Medicine & Public Health, University of California, San Diego, CA, United States of America; 6 Ministerio de Salud Pública y Asistencia Social, Guatemala City, Guatemala; CEA, FRANCE

## Abstract

**Background:**

Dengue, chikungunya, and Zika viruses are increasingly important public health problems. Burning vegetation, leaves, and other plant products have been shown to be effective mosquito repellents for their vector, *Aedes spp*., but there has been scant research on whether firewood cooking smoke in households influences mosquito populations or mosquito-borne diseases. About 2.9 billion people worldwide use biomass fuel for household cooking and heating, resulting in an estimated 1.6 million deaths annually from household air pollution (HAP)-related diseases. Global health agencies now encourage households to transition from biomass to clean fuels, but it is unclear whether such interventions may actually increase risk for mosquito-borne diseases. This retrospective case-control study evaluated associations between arboviral infections and cooking with firewood in Santa Rosa, Guatemala.

**Method:**

*Vigilancia Integrada Comunitaria* (VICo) was a prospective public health surveillance system for bacterial, parasitic, and viral causes of diarrheal, neurological, respiratory, and febrile illnesses in hospitals and clinics in the department of Santa Rosa, Guatemala. Enrolled VICo in-patients and out-patients during 2011–2018 were interviewed using standardized questionnaires on demographics and household characteristics. Blood and stool specimens were collected and tested to identify the etiologies presenting symptoms. Cases were defined as laboratory-positive for dengue, chikungunya, or Zika virus infections. Controls were laboratory-positive for bacterial and viral diarrheal illnesses (e.g., *Salmonella*, *Shigella*, *Campylobacter*, *Escherichia coli*, rotavirus, norovirus, sapovirus, or astrovirus). Cooking with firewood, kitchen location, stove type, and firewood cooking frequency were the independent exposure variables. Logistic regression models were used to analyze unadjusted and adjusted associations between arboviral infections and exposures of interest.

**Result:**

There were 311 arboviral cases and 1,239 diarrheal controls. Arboviral infections were inversely associated with cooking with firewood in the main house (AOR: 0.22; 95% CI: 0.08–0.57), cooking with firewood on an open hearth (AOR: 0.50; 95% CI: 0.33–0.78), and cooking with firewood ≥5 times per week (AOR: 0.54; 95% CI: 0.36–0.81), adjusting for age, sex, ethnicity, socioeconomic status index, number of people per household, community population density, community elevation, recruitment location, season, and admission year.

**Conclusion:**

Several primary determinants of HAP exposure were inversely associated with arboviral infections. Additional studies are needed to understand whether interventions to reduce HAP might actually increase risk for mosquito-borne infectious diseases, which would warrant improved education and mosquito control efforts in conjunction with fuel interventions.

## Introduction

With the emergence and reemergence of dengue (DENV), chikungunya (CHIKV), and Zika (ZIKV), arthropod-borne viruses (arboviruses) are increasingly important public health challenges [1–3]. The first major DENV epidemics were reported in 1779 and 1780 in Africa, Asia, and North America [4]. DENV is now the most prevalent and rapidly spreading of the arboviruses, with transmission occurring in 128 countries, thereby creating risk for almost 4 billion people [5–7]. There are 390 million DENV infections (95% credible interval: 284–528 million) worldwide annually, including 96 million (95% CI: 67–136 million) clinical cases, 500,000 dengue hemorrhagic fever cases, and 22,000 deaths, mostly among children <5 years of age [8]. CHIKV was first reported in the Americas in 2013, causing 1.8 million suspected cases from 2014–2017 in 44 countries and territories [3]. CHIKV may also cause prolonged arthritis, meningoencephalitis, nephritis, retinitis, uveitis, myelitis, cranial nerve palsies, and acute encephalopathy [9]. First identified in Uganda in 1947, ZIKV expanded into the South Pacific and Americas with 48 countries reporting active ZIKV by 2017 and 86 by 2019 [10–12]. From 2015–2018, there were over 580,000 suspected and 220,000 confirmed ZIKV cases in the Americas [2]. ZIKV has also been linked to congenital microcephaly, Guillain-Barré syndrome, craniofacial disproportion, cerebral palsy, spasticity, hearing loss, brainstem dysfunctions, joint deformities, and developmental and inflammatory ocular diseases [11, 13]. This study focused on Guatemala, a country where arboviruses are endemic. Large arbovirus outbreaks have occurred in Guatemala with nearly 40,000 DENV cases from 2014–2015 [14], 57,000 CHIKV cases from 2014–2015 [14], and 4,000 suspected ZIKV cases and 1,000 confirmed cases from 2015–2017 [15].

The most common mode of DENV, CHIKV, and ZIKV transmission is via *Aedes (Ae*.*)* mosquitoes, particularly *Ae*. *aegypti* or *Ae*. *albopictus*. Climate change, urbanization, migration, increased air travel, human behaviors, and ecosystem modification are some factors driving the geographic spread of *Aedes* mosquitoes and their associated viruses [12, 16].

Low socio-economic status (SES) in many settings has been associated with increased risk for arboviral infection [17–19]. Poverty creates ideal conditions for vector proliferation, such as limited access to water infrastructure, garbage disposal services, street drainage, sewage systems, and yard maintenance [20, 21]. It is important to understand the associations between arbovirus transmission and environmental risk factors in order to apply appropriate control measures that may reduce transmission and eliminate arboviruses in endemic areas.

Smoke from burning biomass materials is the most widely used mosquito repellent in the rural tropics [22]. Burning vegetation, leaves, and other plant products have been shown to be effective mosquito repellents [23–27], but there has been scant research on whether smoke from household firewood fires influences mosquito populations, mosquito bites, or mosquito-borne diseases. The few studies of associations between firewood smoke and mosquito abundance are inconsistent. Some studies report firewood smoke reduced *Anopheles* and *Culex* populations in the household resulting in fewer mosquito bites [28–30]. Another study demonstrated inverse associations between firewood smoke and *Aedes* larvae [31]. Other studies were unable to support firewood smoke as an effective mosquito repellent with respect to malaria infection, which is transmitted by *Anopheles* [32–34]. The repellent effect is lost when occupants leave the home and its smoky environment, but smoke residue on skin may provide some repellency by masking human kairomones such as carbon dioxide [22]. We are unaware of any studies assessing the impact of firewood smoke on *Aedes*-transmitted arboviruses. Mosquito repellent benefits from burning firewood are also likely outweighed by other serious health problems from inhaling biomass smoke [35].

About 2.9 billion people worldwide depend on biomass fuel, such as wood, charcoal, coal, animal dung, and crop residues, for their household cooking and heating [36]. However, use of these fuel sources inside houses produces high levels of household air pollution (HAP), including particulate matter, methane, carbon monoxide, polyaromatic hydrocarbons, and volatile organic compounds, which may penetrate into organs and tissue [37]. Exposure to HAP contributes to 1.6 million deaths annually from stroke, ischemic heart disease, chronic obstructive pulmonary disease, and lung cancer [35, 38]. HAP exposure has also been linked with other cancers (e.g., cervical), pneumonia, decreased lung function, adverse pregnancy outcomes, asthma, and cognitive impairment [35, 39]. Consequently, major global health investments are now made to accelerate the transition from biomass to clean fuels. For example, the Global Alliance for Clean Cookstoves is working to reduce the use of fuel burning stoves and to increase the number of improved cook stoves in Guatemala [40]. Given the growing public health importance of arboviruses in the Americas, it is important to understand whether such interventions might have unintended consequences, such as increasing risks for mosquito-borne infectious diseases. This study focuses on firewood, which is the predominant energy source for cooking in Central America [41]. To our knowledge, this is the first study to investigate associations between DENV, CHIKV, or ZIKV infection and household air pollution (HAP) or specific characteristics of firewood cooking in the household. This study evaluates the associations of cooking with firewood, the location in the house where someone cooks with firewood, the type of stove used to cook with firewood, and the times per week cooking at home with firewood, with arboviral infections in Santa Rosa, Guatemala, where firewood cooking is the most common cooking method [40].

## Materials and methods

### Study design

*Vigilancia Integrada Colaborativa* (VICo) was a prospective public health sentinel surveillance system for bacterial, parasitic, and viral causes of diarrheal, neurological, respiratory, and febrile illnesses in Guatemala. Hospital surveillance began in Cuilapa, Santa Rosa, in November 2007. Health center surveillance began in Nueva Santa Rosa Municipality in July 2007. We conducted a retrospective case-control study to examine associations between *Ae*. *aegypti*-transmitted arboviruses (DENV, CHIKV, or ZIKV infection) and HAP exposure. Additional details of VICo methodology are described elsewhere [42–46].

### Study setting

**Fig 1:**
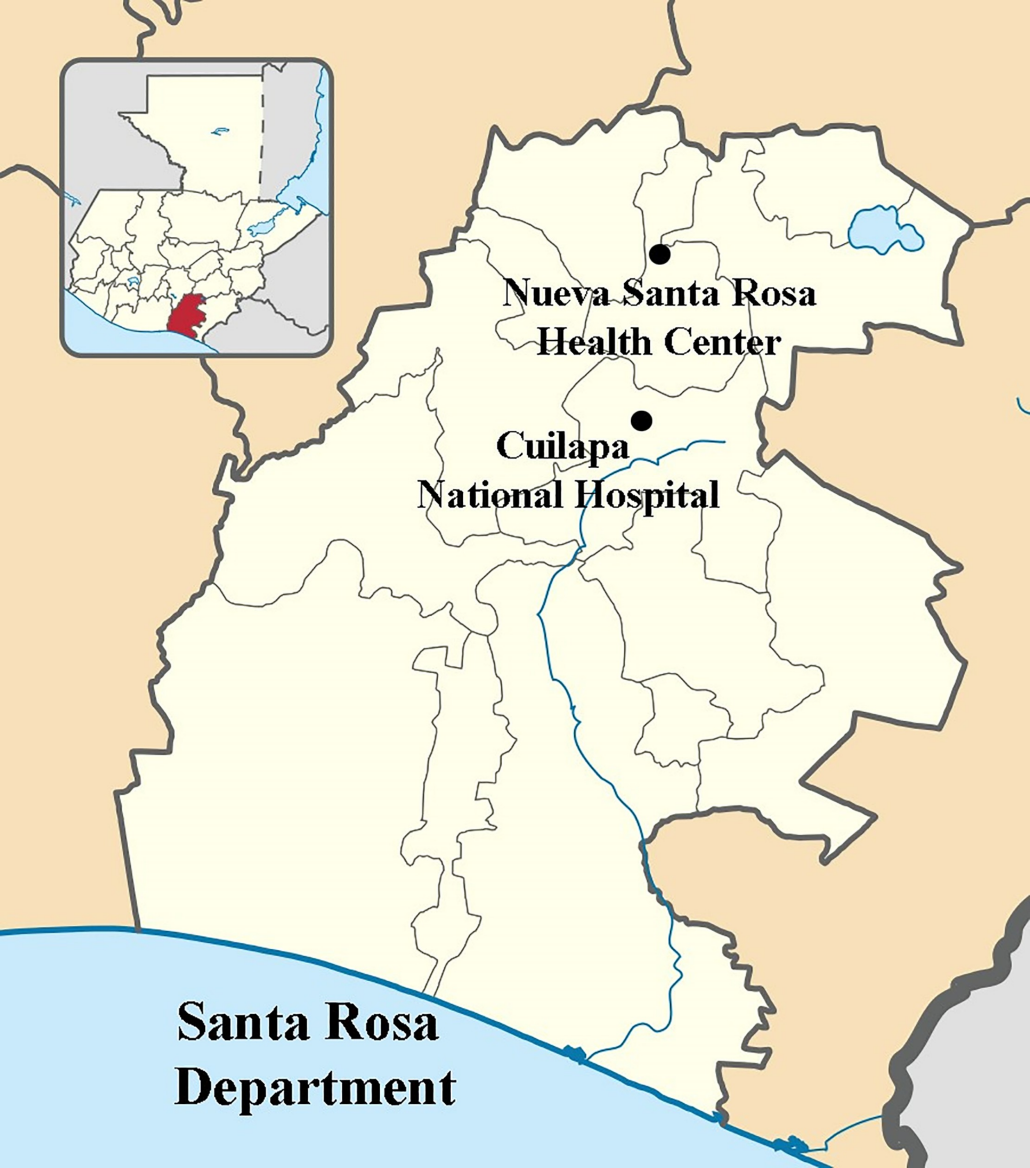
Cuilapa national hospital and nueva Santa Rosa health center, Santa Rosa department, Guatemala. Source: Santa Rosa department location map; by user Edgouno; licensed under CC BY 3.0 via Wikimedia Commons, https://commons.wikimedia.org/wiki/File:Quetzaltenango_department_location_map.svg.

The Department of Santa Rosa, Guatemala, (14.16°N, 90.48°W) has a population of approximately 400,000 in an area of 2,295 km^2^ and is semi-tropical (Fig 1) [47]. Its altitude varies from sea level on the Pacific Coast to 1,945 m on top of the Tecuamburro volcano. The mean annual temperature is 23.5°C and mean annual precipitation is 1,412 mm [48]. The population of the Department is 55% rural and 45% urban and is almost equally divided between women and men [49]. Countrywide, 2.1 million households (59.7%) use wood fuel, including 1.3 million in rural and 0.8 million in urban areas [40, 49]. In 2013, 97% of rural and 85% of urban residences used firewood for fuel in Santa Rosa [40].

The National Hospital of Cuilapa serves all 400,000 residents from Santa Rosa as well as referrals from municipalities of neighboring Departments of Jutiapa and Jalapa. VICo also included a health center in Nueva Santa Rosa municipality, located 30 km north of Cuilapa [43].

### Study population

Inclusion criteria for the study included residency in Santa Rosa, Jutiapa, and Jalapa Departments during the 30 days before presenting to the National Hospital of Cuilapa or health center in the Nueva Santa Rosa municipality. All ages and both males and females were included.

At the hospital and health center, surveillance staff searched the emergency room records, inpatient logs, and ward registers to identify patients admitted or presenting with signs or symptoms suggestive of acute febrile illnesses (AFI) or diarrhea. AFI was defined as self-reported fever that began within one week of the current illness, or documented oral or axillary fever of ≥38ºC at presentation or within 24 hours of admission to the hospital or health center. Patients with evidence of an obvious source of fever on physical examination (e.g., otitis media, septic arthritis, pyogenic soft tissue infection) determined by the examining healthcare provider were excluded. Diarrhea was defined as ≥3 loose or liquid stools in a 24-hour period with onset of illness within seven days before presenting to the hospital or health center. Patients with other diarrheal episodes in the week before the start of the current episode were excluded to avoid capturing illnesses due to chronic diarrhea.

Following informed consent, trained surveillance nurses administered face-to-face interviews to all patients who met the case definitions and agreed to participate or their parents/guardians. Handheld personal digital assistants were used to collect demographic, epidemiologic (including firewood cooking variables), and socioeconomic data. This survey interview was only conducted once. Immediately thereafter, clinical data was abstracted from medical charts and appropriate laboratory samples were collected to determine the infection etiology.

Four ml of whole blood were collected from hospitalized patients with AFI, which were transported to the Universidad del Valle de Guatemala (UVG) laboratory. Plasma was separated and frozen at -70°C. Reverse transcription polymerase chain reaction (RT-PCR) (DENV, CHIKV, ZIKV) and enzyme-linked immunosorbent assay (ELISA) IgM (DENV) tests were done at the UVG laboratory to confirm arboviral infection. Patients with AFI enrolled in the study were not tested for diarrheal illnesses.

A bulk stool specimen was collected from all consenting subjects enrolled with diarrhea. Rectal swabs were done on children <5 years of age who were unable to provide a specimen. Stool culture was used to detect bacterial infections including *Salmonella* spp., *Shigella* spp., and *Campylobacter* sp. ELISA IgM was used to detect rotavirus. RT-PCR was used to detect norovirus-1 and 2, sapovirus, and astrovirus. Conventional PCR was used to detect *Escherichia coli*. Due to relatively low sensitivity of fecal smear microscopy [50–52] and weak associations with diarrheal illnesses [53], parasitic infections (e.g., *Cryptosporidium parvum*, *Giardia lamblia*) were excluded from analyses. Patients enrolled into the study with diarrheal symptoms were not tested for arboviruses due to the enrollment and testing procedures for the surveillance system (at enrollment into the study, patients were assigned to febrile or diarrheal groups based on their symptoms and laboratory tests were conducted accordingly).

Cases were defined as those presenting AFI who tested positive for DENV, CHIKV, or ZIKV infection based on RT-PCR or ELISA tests from 2011 (when the questions regarding cooking with firewood were added to the survey) to 2018. Controls were confirmed bacterial and viral diarrheal illnesses during 2011–2018 (e.g., *Salmonella* spp., *Shigella* spp., *Campylobacter* sp., *Escherichia coli*, rotavirus, norovirus-1 and 2, sapovirus, and astrovirus). We only included diarrheal controls with confirmed bacterial or viral infections because diarrhea may be a symptom of an arbovirus infection [54]. We did not exclude any arbovirus cases nor any controls diagnosed with any of the specific pathogens of interest.

### Ethics statement

The protocol for the VICo surveillance project received approval from the institutional review boards of UVG (Guatemala City, Guatemala), the Centers for Disease Control and Prevention (Atlanta, GA, USA), and the Guatemala Ministry of Public Health and Welfare. This study uses de-identified data from VICo and was determined to qualify for exemption from full Institutional Review Board review. Patients were asked for verbal consent for eligibility screening. If they satisfied the case definitions, they were asked for written, informed consent for participation in the study. We obtained verbal assent from minors <18 years of age and written, informed consent from their parents or legal guardians.

### Exposure and covariate measures

The key HAP exposure classifications were: firewood is used as the main fuel for cooking in household, location in the house where firewood cooking is done, type of stove used to cook with firewood, and times per week cooking at home with firewood.

We used principal components analysis (PCA) to create a HAP exposure score from firewood cooking location in house, firewood stove type, and firewood cooking frequency based on all cases and controls (S1 Table). First, we assigned scores ranging from 0–3 based on HAP exposure levels: firewood cooking location (3: in the main house, 2: in a kitchen that is separated from the main house or in an informal structure without walls or roofs, 1: outside the house, 0: does not cook with firewood), type of stove used to cook with firewood (3: open hearth, 2: improved stove without chimney, 1: improved stove with chimney, 0: does not cook with firewood), and times per week cooking at home with firewood (3: ≥5, 2: 3–4, 1: 1–2, 0: does not cook with firewood). The first component included all three variables and accounted for 88% of the variability in the data, and these variables were then weighted against their eigenvector coefficients [55]. The resultant score was categorized into quintiles of HAP exposure levels (very low, low, middle, high, very high).

Covariates included year of admission (2011–2018), season (dry, rainy), age (continuous), sex (female, male), ethnic group (Ladino, Xinca, other), recruitment location (hospital, health center), number of people in the house (continuous), community elevation (continuous), and community population density (continuous). The ‘Ladino’ people are Central Americans with a mix of indigenous and Spanish descent. The ‘dry’ season was from November-May, whereas ‘rainy’ was from June-October. Geographical information system software (ArcGIS Pro 2.2.4 software; ESRI, Redlands, CA) was used to calculate average elevation (meters) and average population density (numbers of people per hectare) per community. Population densities were obtained from WorldPop 2015 [56]. Elevations were from the Consortium for Spatial Information (CGIAR-SRTM) [57]. PCA was used to create a SES index from 14 variables based on all cases and controls and included: presence of a refrigerator, computer, radio, washing machine, clothes dryer, car, television, telephone/cellphone, microwave; number of rooms in house; family monthly income; electricity; roof material; and floor material (S1 Table). Missing data for SES variables were assigned the lower category. One component was developed and retained which accounted for 29% of the variability in the data. Other components explained little variability in the data. These variables were weighted against their eigenvector coefficients. The SES index was categorized into quintiles with scores ranging from 0 to 5 with a higher score indicating higher SES.

### Statistical analysis

Exposures and covariates for cases and controls were first evaluated with descriptive statistics (means and standard deviations for continuous variables and frequency distributions for categorical variables). T-tests and Chi-square tests were then used to assess differences between cases and controls for continuous and categorical variables, respectively. The Chi-square test for trend (extended Mantel–Haenszel) was used to test linear trends in HAP scores between cases and controls.

Logistic regression was used to analyze unadjusted (Model 1) and adjusted (Model 2) associations between arboviral infections and exposures of interest (cooks with firewood, firewood cooking frequency, kitchen location, stove type, and HAP score). In Model 2, confounders were identified *a priori* from the literature using directed acyclic graphs [58, 59]: age [18, 19], sex [60], ethnic group [61], SES index [18, 62], admission year [63], season [63], number of people in household [64], population density [65], recruitment location, and elevation [66]. We chose not to match on age, location, and year to prevent biases associated with matching in case-control study designs [58, 67]. We considered linear, quadratic, and cubic forms of age and SES index. Since most of the cooking in Guatemala is done by women [40], we also looked to see whether there were interactions between sex and the exposures of interest on arboviral infections in Model 2. Tolerance values were used to assess collinearity among all independent variables. Hosmer-Lemeshow tests were used to assess goodness-of-fit of adjusted models. Odds ratios (OR) described the magnitude of associations between arboviral infection and exposures of interest. Statistical significance, defined as p<0.05, was evaluated through the Chi-square test. ORs, 95% confidence intervals, and p-values were reported. All analyses were conducted using SAS V.9.4 (SAS Institute, Inc., Cary, North Carolina).

## Results

### Sample characteristics

Of 854 total febrile illnesses identified through the hospital and health center surveillance system, there were 311 arbovirus cases (219 DENV, 75 CHIKV, and 29 ZIKV infections). Twelve (3.9%) had dual infections. Of the 3,719 diarrheal illnesses identified through the surveillance system, there were 1,239 patients with the specific pathogens included in the study design (Table 1). Of arbovirus cases and diarrheal controls, 199 and 750 were recruited from the hospital, whereas 112 and 489 were recruited from the health center, respectively (Table 2).

**Table 1:** Arbovirus frequency among cases; viral and bacterial infections among diarrheal controls, Santa Rosa, Guatemala, 2011–2018.

	N (%)
*Cases (N = 311)*^*a*^	
Dengue	219 (70.4)
Chikungunya	75 (24.1)
Zika	29 (9.3)
*Controls (N = 1*,*239)*^*b*^	
*Salmonella* spp.	13 (1.1)
*Shigella* spp.	144 (11.6)
*Campylobacter* sp.	190 (15.3)
*Escherichia coli*	326 (26.3)
Astrovirus	49 (4.0)
Sapovirus	49 (4.0)
Norovirus-1	48 (3.9)
Norovirus-2	388 (31.3)
Rotavirus	290 (23.4)

^a^Cases may have been diagnosed with multiple arboviruses (3.9%).

^b^Controls may have been diagnosed with multiple viral and/or bacterial infections among those listed (20.8%).

**Table 2:** Cases with confirmed arbovirus infection (dengue, chikungunya, or Zika virus) and controls with confirmed diarrheal infections^a^, Santa Rosa, Guatemala, 2011–2018.

	Cases	Controls	
Characteristic	N = 311	N = 1,239	p-value^b^
*Categorical variables (n and %)*			
Cooks with firewood			**<0.001**
Yes	207 (66.6)	932 (75.2)	
No	104 (33.4)	307 (24.8)	
Times per week cooking at home with firewood			**<0.001**
≥5	168 (54.0)	837 (67.5)	
3–4	14 (4.5)	42 (3.4)	
1–2	25 (8.1)	53 (4.3)	
Does not cook with firewood	104 (33.4)	307 (24.8)	
Location in house where patient cooks with firewood			**<0.001**
In main house	6 (1.9)	82 (6.6)	
In a kitchen that is separated from main house	158 (50.8)	615 (49.6)	
In an informal structure without walls/roofs	24 (7.7)	137 (10.6)	
Outside the house	19 (6.1)	104 (8.4)	
Does not cook with firewood	104 (33.5)	307 (24.8)	
Type of stove used to cook with firewood			**<0.001**
Open hearth fire	98 (31.5)	656 (52.9)	
Improved stove without chimney	66 (21.2)	135 (10.9)	
Improved stove with chimney	43 (13.8)	141 (11.4)	
Does not cook with firewood	104 (33.4)	307 (24.8)	
HAP score^c^			**<0.001**
Very high	3 (1.0)	67 (5.4)	
High	61 (19.6)	449 (36.2)	
Middle	76 (24.4)	230 (18.6)	
Low	67 (21.5)	186 (15.0)	
Very low	104 (33.5)	307 (24.8)	
Sex			**0.036**
Female	161 (51.8)	559 (45.1)	
Male	150 (48.2)	680 (54.9)	
Ethnic group			**<0.001**
Ladino	198 (63.7)	985 (79.5)	
Xinca	103 (33.1)	207 (16.7)	
Other	10 (3.2)	47 (3.8)	
Recruitment location			0.269
Hospital	199 (64.0)	750 (60.5)	
Health center	112 (36.0)	489 (39.5)	
Season			0.546
Dry	108 (34.7)	408 (32.9)	
Rainy	203 (65.3)	831 (67.1)	
Admission year			**<0.001**
2011–2012	5 (1.6)	302 (24.4)	
2013–2014	42 (13.5)	359 (29.0)	
2015–2016	247 (79.4)	383 (30.9)	
2017–2018	17 (5.5)	195 (15.7)	
*Continuous variables (median and IQR)*			
Age	22.6 (12.7–40.0)	18.2 (10.2–32.4)	**<0.001**
Number of people per household	5 (4–6)	5 (4–6)	0.557
Socioeconomic status index^d^	2.1 (1.5–2.7)	1.7 (1.2–2.4)	**<0.001**
Number of people per hectare per community	2.5 (2.0–2.8)	2.6 (2.1–2.9)	0.053
Community elevation (m)	1,043 (941–1,217)	1,150 (1,098–1,232)	**0.006**

HAP: household air pollution, IQR: interquartile range

^a^ *Salmonella* spp., *Shigella* spp., *Campylobacter* sp., *Escherichia coli*, rotavirus, norovirus-1 and 2, sapovirus, and astrovirus.

^b^ Categorical variables: p-value from chi-square test; continuous variables: p-value from t-test

^c^ HAP score was derived from principal components analysis and included: firewood cooking frequency, firewood cooking location, and stove type. The chi-square test for trend (extended Mantel–Haenszel) was used to test linear trends in HAP scores between cases and controls.

^d^ Socioeconomic status index was derived from principal components analysis and included: a refrigerator, computer, radio, washing machine, dryer, car, television, phone, and microwave; number of rooms in house; income; electricity; roof and floor material. Score range: 0 to 5

The average age of patients was 21 years, 54% were male, and 76% were Ladino ethnicity (Table 2). Of cases and controls, 67% and 75% respectively cooked with firewood, 54% and 68% cooked with firewood at least five times per week, 32% and 53% cooked with firewood on an open hearth, and 2% and 7% cooked with firewood in the main house. Among all study participants who did not cook with firewood, 98% cooked with gas and 2% cooked with electricity.

### Arboviral infection and cooking with firewood

Unadjusted analyses demonstrated inverse associations between arboviral infections and cooking with firewood (OR: 0.66; 95% CI: 0.50–0.86); cooking with firewood ≥5 times per week (OR: 0.59; 95% CI: 0.45–0.78), in the main house (OR: 0.22; 95% CI: 0.09–0.51), in an informal structure without walls/roofs (OR: 0.54; 95% CI: 0.33–0.88), outside the house (OR: 0.54; 95% CI: 0.32–0.92), and on an open hearth (OR: 0.44; 95% CI: 0.32–0.60); and high HAP score (OR 0.40; 95% CI: 0.28–0.57) and very high HAP score (OR 0.13; 95% CI: 0.04–0.43) (Table 3).

**Table 3:** Unadjusted and adjusted^a^ associations between arboviral infection (dengue, chikungunya, or Zika virus) and indicators of household air pollution exposure, Santa Rosa, Guatemala, 2011–2018 (N = 311 cases and 1,239 controls^b^).

Characteristic	OR (95% CI)	AOR^a^ (95% CI)
Cooks with firewood	**0.66 (0.50**–**0.86)**	0.71 (0.47–1.07)
Does not cook with firewood	REF	REF
Times per week cooking at home with firewood		
≥5	**0.59 (0.45–0.78)**	**0.54 (0.36–0.81)**
3–4	0.98 (0.52–1.87)	1.27 (0.56–2.87)
1–2	1.39 (0.82–2.35)	1.08 (0.57–2.05)
Does not cook with firewood	REF	REF
Location where patient cooks with firewood		
In main house	**0.22 (0.09–0.51)**	**0.22 (0.08–0.57)**
In a kitchen that is separated from main house	0.76 (0.57–1.01)	0.73 (0.48–1.09)
In an informal structure without walls/roofs	**0.54 (0.33–0.88)**	0.58 (0.31–1.10)
Outside the house	**0.54 (0.32–0.92)**	0.60 (0.31–1.15)
Does not cook with firewood	REF	REF
Type of stove used to cook firewood		
Open hearth fire	**0.44 (0.32–0.60)**	**0.50 (0.33–0.78)**
Improved stove without chimney	1.44 (1.00–2.09)	1.24 (0.72–2.13)
Improved stove with chimney	0.90 (0.60–1.35)	0.67 (0.40–1.12)
Does not cook with firewood	REF	REF
HAP score^c^		
Very high	**0.13 (0.04–0.43)**	**0.12 (0.03–0.44)**
High	**0.40 (0.28–0.57)**	**0.41 (0.25–0.68)**
Middle	0.97 (0.69–1.37)	1.00 (0.61–1.63)
Low	1.06 (0.74–1.52)	0.76 (0.48–1.20)
Does not cook with firewood	REF	REF

CI: confidence interval; OR: odds ratio; AOR: adjusted odds ratio; HAP: household air pollution

^a^Adjusted for linear age, sex, ethnic group, admission year, season, number of people in household, recruitment location, community population density, community elevation, and linear socioeconomic status index. Socioeconomic status index was derived from principal components analysis and included: a refrigerator, computer, radio, washing machine, dryer, car, television, phone, and microwave; number of rooms in house; income; electricity; roof and floor material.

^b^Diarrheal illnesses included *Salmonella* spp., *Shigella* spp., *Campylobacter* sp., *Escherichia coli*, rotavirus, norovirus-1 and 2, sapovirus, and astrovirus.

^c^HAP score was derived from principal components analysis and included firewood cooking frequency, firewood cooking location, and stove type.

Arboviral infections were no longer associated with overall cooking with firewood, cooking with firewood in an informal structure, and cooking with firewood outside, after adjusting for age, sex, ethnicity, SES index, number of people per household, community population density, community elevation, recruitment location, season, and admission year (Table 3).

Even after adjustment, analyses showed associations between arboviral infections and the two highest level HAP exposure classifications (high HAP scores: AOR: 0.41; 95% CI: 0.25–0.68; very high HAP scores: AOR: 0.12; 95% CI: 0.03–0.44). Arboviral infections remained inversely associated with cooking with firewood ≥5 times per week (AOR: 0.54; 95% CI: 0.36–0.81), cooking with firewood in the main house (AOR: 0.22; 95% CI: 0.08–0.57), and cooking with firewood on an open hearth (AOR: 0.50; 95% CI: 0.33–0.78), even after adjusting for relevant covariates (Table 3). Tolerance values for all independent variables were above 0.90, indicating no evidence of collinearity. Interaction terms between sex and exposures of interest were not significant.

## Discussion

Results of our case-control study suggest that arboviral infections were inversely associated with exposure to higher levels of biomass smoke. We did not find associations between arboviral infections and lower levels of biomass smoke. These results were supported by analyses that treated HAP exposure as a composite score, which demonstrated inverse associations with the two highest HAP exposure levels.

The odds of cooking with firewood in the main house were less among patients with arboviral infections than among controls. HAP exposure is likely higher in households with kitchens in the main household rather than in a separate location [68]. We did not find associations between arboviral infections and cooking with firewood outside or in a kitchen separate from the main house, implying that any arboviral-protective benefit from smoke exposure might be limited to confined household spaces. In Guatemala, approximately 90% of urban households and 70% of rural households conduct cooking activities inside the main house [40]. Previous studies have demonstrated that firewood smoke was effective at reducing the number of *Anopheles spp*. found in households [28, 30]. To our knowledge, only two studies have assessed kitchen location in relation to mosquitoes or mosquito-borne diseases. In Laos, cooking fires in the main living area or directly underneath houses were associated with fewer *Anopheles spp*. than fires in rooms separate from the house [30]. In Ethiopia, individuals living in households that had a separate kitchen outside of the sleeping room were at greater risk for malaria [69]. However, we are unaware of any studies examining the impact of cooking with firewood on arboviral infections. In Guatemala, cooking activities are carried out for approximately 13 and 14 hours per week in urban and rural areas, respectively [40]. Additionally, women spend 4.6–6.8 hours in the kitchen per day [70]. Cooking activities, which are mostly done in the daytime [40], likely have a differential effect on *Ae*. *aegypti*, which are primarily daytime feeders compared to *Anopheles spp*., which are nocturnal but may include crepuscular feeders [71–73]. *Ae*. *aegypti* preferentially rest indoors in dark places (e.g., on walls, in closets, or underneath furniture) and lay eggs in artificial containers around households [74], whereas *Anopheles spp*. rest indoors and outdoors, but prefer marshes, trees, swamps, fields, streams, and rivers as oviposition sites [75]. *Ae*. *aegypti* are also more likely to bite indoors than outdoors [76], whereas *Anopheles spp*. mostly bite outdoors [77].

Cooking with firewood ≥5 times per week was less common among arbovirus cases than among controls, but there was no association with cooking with firewood <5 times per week. These findings are consistent with a study in rural Thailand that found inverse associations between firewood smoke and *Aedes* larvae abundance [31]. Firewood cooking produces biomass smoke, which may influence mosquitoes by masking human odors such as carbon dioxide [22], interfering with mosquito chemoreceptors [78], or emitting organic compounds that serve as insecticides [22, 34]. Alternatively, the heat from firewood cooking may reduce room humidity, creating an unfavorable environment for mosquitoes [28]. Stove use monitoring could improve understanding of the relationships between cooking frequency and arboviral infections.

Arboviral infections were lower among patients who cooked on an open hearth, but there was no association observed with cooking with improved stoves. Chimney stoves reduce kitchen concentrations of carbon monoxide and particulate matter by approximately 90% and personal exposures in women by 61% [79], but may also inadvertently increase exposure to mosquitoes in the household [34, 80]. Additional studies are needed to determine whether HAP interventions should be combined with mosquito prevention strategies [34]. Insecticide-treated bed nets, window screens, protective clothing, and air conditioning are safe and effective arbovirus prevention measures [81–83].

The interaction between gender and firewood cooking on arboviral infections did not reach statistical significance. Cooking is mainly done by women in Guatemala [40], but it is conceivable that men are present in the household during cooking activities. It is also possible that we did not have adequate power to detect a gender-related interaction.

Three-quarters of cases and two-thirds of controls cooked with firewood, which is higher than the prevalence of firewood use in all of Guatemala (59.7%). This difference may be attributed to the high proportion of rural residences in Santa Rosa Department (58.1%), as well as the high prevalence of firewood use in Santa Rosa Department (rural households: 97%; urban households: 85%) [40].

This study has several limitations. First, there is likely unmeasured confounding in this study, such as whether participants used mosquito prevention measures (e.g., mosquito nets, fumigating), the number of open water-holding containers around patients’ households, household sanitation, and arbovirus transmission site. It could be that cases were infected at work, school, or elsewhere away from the home where they were not exposed to firewood smoke. However, we do not expect other sources of smoke to be strong confounders, and our adjustment for community population density should help reduce biases associated with arbovirus transmission sites. Second, this was a case-control study, so we are unable to make causal inferences about the relationship between arboviral infection and firewood cooking, only associations. Third, this study included patients from a hospital and health center and is thus not representative of all of Santa Rosa Department or Guatemala. Fourth, although we only included diarrheal controls with confirmed bacterial or viral infections in an attempt to ensure controls were not cases, most diarrheal controls were not tested for arboviruses. Non-differential misclassification of the outcome may dilute the magnitude of the odds ratios (biased towards the null). Fifth, we do not know when arbovirus transmission occurred in relation to wood smoke exposure, but our questionnaire reflects the patients’ typical past exposures. Sixth, it is unknown whether hospital controls with diarrheal illnesses, like norovirus, influenced the susceptibility of the patients to arboviruses. Finally, it is unknown whether HAP exposure influences diarrheal controls’ susceptibility to a diarrheal infection and therefore, selection into this study (Berkson’s bias). However, one study in California demonstrated PM_10_, COH, NO_2_, and O_3_ were not associated with gastroenteritis [84]. An attempt was made to minimize this risk by limiting the controls to confirmed bacterial and viral diarrheal illnesses, and excluding respiratory infections and undiagnosed diarrheal illnesses. There were insufficient numbers of febrile illnesses of other infections (e.g., *Leptospira*) and neurological illnesses to serve as an additional control group.

Notwithstanding these limitations, this study included approximately four controls per case, which increased statistical precision. We assessed multiple measures of HAP exposure, including household kitchen location, firewood cooking frequency, and stove type and found associations that strengthened with increased HAP exposure. The interviewers collecting HAP exposure data were unaware that HAP might to be associated with arbovirus infections. Controls were recruited from the same catchment area as cases.

HAP exposure is a major risk factor for acute and chronic respiratory diseases. Particulate matter exposure risks include respiratory symptoms; acute and chronic decrement in pulmonary function; bronchial hyperactivity; acute phase reaction; respiratory infections, emergency department visits, and hospitalizations; asthma development; and premature mortality in people with chronic lung disease [35, 39]. We found anecdotal evidence that suggests households that frequently cook with firewood may have fewer arboviral infections than households that do not cook with firewood. Rather than suggesting that biomass smoke be employed as a preventive measure, these findings suggest that arboviral surveillance studies should monitor levels and trends during efforts to reduce HAP exposures in order to help determine whether a causal relationship exists. Given the public health importance of arboviruses in the Americas, it is important to understand whether interventions to reduce HAP might actually increase risks for mosquito-borne infectious diseases—especially during transmission season or outbreak periods, which would warrant expanded education and vector control efforts in conjunction with interventions to reduce HAP.

## Supporting information

S1 Table Principal components analysis of socioeconomic and household air pollution variables, Santa Rosa, Guatemala (n = 1,550). (DOCX)
