# Supplementary material for: Inverse association between dengue, chikungunya, and Zika virus infection and indicators of household air pollution in Santa Rosa, Guatemala: A case-control study, 2011-2018
Source: PLoS One. 2020 Jun 19;15(6):e0234399. doi: 10.1371/journal.pone.0234399 (PMC7304608; doi:10.1371/journal.pone.0234399)
Supplement: S1 Table — (DOCX) [file pone.0234399.s001.docx]

| **S1 Table. Principal components analysis of socioeconomic and household air pollution variables, Santa Rosa, Guatemala (n=1,550)** | |
| --- | --- |
| Characteristic | Eigenvector |
| **Socioeconomic status index** |  |
| Car | 0.31 |
| Computer | 0.32 |
| Microwave | 0.30 |
| Radio | 0.25 |
| Refrigerator | 0.38 |
| Telephone | 0.18 |
| Television | 0.31 |
| Washing machine | 0.29 |
| Dryer | 0.10 |
| Electricity | 0.24 |
| Number of rooms | 0.24 |
| Roof type | 0.13 |
| Floor type | 0.27 |
| Income | 0.30 |
| *Eigenvalue* | 3.63 |
| **Household air pollution score** |  |
| Firewood cooking frequency | 0.59 |
| Firewood cooking location | 0.58 |
| Stove type | 0.56 |
| *Eigenvalue* | 2.64 |
